# Quantifying the broader economic consequences of quadrivalent human papillomavirus (HPV) vaccination in Germany applying a government perspective framework

**DOI:** 10.1186/s13561-015-0054-6

**Published:** 2015-07-22

**Authors:** Nikolaos Kotsopoulos, Mark P Connolly, Vanessa Remy

**Affiliations:** 1 Department of Pharmacy, Unit of PharmacoEpidemiology & PharmacoEconomics, University of Groningen, Antonius Deusinglaan 1, 9713 AV Groningen, The Netherlands; 2 Global Market Access Solutions (GMAS), St-Prex, Switzerland; 3 Sanofi Pasteur MSD, Lyon, France

**Keywords:** Human papillomavirus, Fiscal analysis, Cost-benefit analysis, Vaccination, Germany, Microeconomics, Lifetime modelling, H7, H51, H57, I12, I18

## Abstract

HPV infections can cause substantial burden in females and males as it is associated with several genital cancers, in addition to genital warts. Traditional economic evaluations often focus on quantifying cost-effectiveness, however, it is increasingly recognized that vaccinations may generate broader benefits not captured in cost-effectiveness analysis. Τhe aim of this study was to evaluate the broader economic consequences associated with HPV vaccination in males and females and to conduct a lifetime cost-benefit analysis of investing in universal vaccination in Germany from the perspective of government. Methodologies from generational accounting, human capital and health economics were combined to estimate the broader economic consequences of HPV vaccination including the fiscal impact for the government. A cohort model was developed simulating the medical costs and average lifetime fiscal transfers between the government and 12-year-old immunized and non-immunized males and females. To estimate tax revenue attributed to vaccination-related changes in morbidity and mortality, direct and indirect tax rates were linked to differences in age- and gender-specific earnings. Based on HPV vaccination costs, the base case cost-benefit analysis demonstrated that investing €1 in universal HPV vaccination could yield €1.7 in gross tax revenue over the lifetime of the cohorts. After taking into consideration the governmental transfers, universal HPV vaccination in Germany could result in incremental positive net discounted taxes (i.e. tax revenue-transfers) from €62 million for the German government. The vaccination of males and females with the quadrivalent HPV vaccine is likely to have positive effects on public finances.

**Electronic supplementary material:**

The online version of this article (doi:10.1186/s13561-015-0054-6) contains supplementary material, which is available to authorized users.

## Backgound

The human papillomavirus (HPV), in particular subtypes 6, 11, 16 and 18, is responsible for a number of conditions in both males and females including genital warts (GW) as well as vaginal, vulvar, cervical and anal cancers in females and penile and anal cancers in males [1]. In Germany, previous studies reported that there are 6,190 new cases every year and about 1,660 deaths [2]. In addition, a broad range of short and long-term direct and indirect medical costs are attributed to HPV encompassing costs for treating genital warts, cervical cancer and other HPV-related cancers [3, 2, 4, 5]. A quadrivalent (6/11/16/18) and a bivalent (16/18) HPV vaccines are available. The quadrivalent vaccine is indicated in males and females from the age of nine years to protect against HPV6/11/16/18 related precancerous lesions in the cervix, vulva, vagina and anus, cervical and anal cancers and genital warts.

In Germany, since 2007, HPV vaccination has been recommended and funded for all females aged 12 to 17, in combination to a yearly cervical cancer screening starting from age 20. Several cost-effectiveness analyses suggested that vaccinating females against HPV is a cost-effective strategy both in Germany and elsewhere [2, 6]. Preventing the long-term mortality and morbidity of HPV infections will result not only in public health benefits, but is also expected to result in considerable economic benefits in terms of medical cost-savings, increased productivity, increased earnings and increased tax revenue for the government.

The acquisition of vaccines for national vaccination programs are unique among healthcare purchases in that stakeholders often include treasury and other federal ministries necessary for procurement decisions [7, 8]. To inform stakeholders of the broader economic consequences of vaccination, there is some potential value to interpret the broader benefits of vaccine investments in terms of future economic growth and fiscal benefits [9]. Τhe aim of this study was to estimate both the broader economic consequences associated with HPV vaccination in males and females in Germany and to conduct a cost-benefit analysis (CBA) of investing in vaccination from a government (or fiscal) and a societal perspective. The emphasis was put on the economic benefits that the government is expected to derive from decreased mortality and morbidity i.e. tax revenue from the higher quantity of survival and reduced health care costs stemming from decreased morbidity.

## Methods and data

To estimate the broader economic consequences of HPV vaccination in males and females we estimated how resulting changes in HPV related morbidity and mortality may influence government fiscal accounts based on the efficacy profile for the quadrivalent HPV vaccine under a fixed economic scenario (“ceteris paribus”). Three analytic frameworks stemming from the health economic, economic and public economic theory were employed to quantify the economic impact of immunization and to compare the benefits and costs associated with HPV vaccination in Germany. The analytic frameworks utilized were health-care budget impact analysis, human capital economics, fiscal modelling and generational accounting. By combining these frameworks it was possible to estimate how preventable HPV-related morbidity and mortality would generate both short and long-term societal and fiscal consequences for the German government.

The health-care budget impact analysis estimated how changes in HPV related mortality and morbidity influence short term and long term health-care public spending. The lifetime human capital of immunized and non-immunized male and female cohorts was projected to determine how changes in HPV related morbidity and mortality affect lifetime earnings. The government direct and indirect tax burden was then applied to wages to determine the expected fiscal revenues in terms of gross tax. Following the principles of generational accounting (Auerbach, 1999) the average lifetime transfer costs and tax revenues attributed to changes in morbidity and mortality resulting from HPV vaccination were also estimated. To avoid double calculations of health-care costs, non-HPV related health expenditure was not included in the analysis. In addition, educational costs were not considered in this analysis since the level of mortality and morbidity during the first years of life for both immunized and non-immunized cohorts were considered as similar. For the three analytic approaches, only those factors that have direct influence on government fiscal accounts were assessed.

The analysis was conducted for a single cohort of 12 year old males and females. The latter age cohort was analyzed in order to quantify the benefits and costs for the German government from the prospective annual vaccination against HPV. Hence, this study economically assesses the hypothesis that, according to the German vaccination guidelines, each year the cohort of males and females that enters the age of 12 will be included in a universal vaccination program.

### Epidemiological modelling

A prospective single-cohort model was developed in Microsoft Excel. The model simulated the lifetime of 12 year old males and females with and without vaccination against HPV. Survival was projected based on lifetable methods hence, based on the current life expectancy in Germany [10]. The cohort model projected the lifetime survival of equally-sized male and female birth cohorts with and without vaccination against HPV. An incident based approach rather than a prevalence based approach was used since only a single cohort of 12 year old males and females was prospectively modelled. Age-specific incidence and age-specific mortality of HPV-related diseases was used to calculate the HPV attributable mortality and morbidity. To calculate the morbidity and mortality of the immunized male and female cohorts, age– and gender–specific incidence and mortality was adjusted for the efficacy of the quadrivalent vaccine against each of the scope HPV-related cancers and pre-cancer states (Table 1). A mathematical illustration of the epidemiological calculations is presented in the Additional file 1: Technical appendix. The following HPV-related diseases were analyzed: genital warts (GW); cervical intraepithelial Neoplasia (CIN) I, II and III; cervical cancer; anal cancer; vulvar cancer and vaginal cancer (head and neck cancers evaluated in sensitivity analysis). The prospective single-cohort model estimated the incremental benefits of vaccination from different perspectives (i.e. governments’ and societal).

**Table 1:** Modelled vaccine’s efficacy per disease and costs per case

Disease	Vaccine efficacy^a^ (1)	Proportion attributable to HPV 6/11/16/18^b^ (2)	Model’s efficacy (1) × (2)	Cost per case	
GW	Females 99 %; 89 % for males	90 %	Females 89 %; males 81 %	€550	[2]
CIN I	98 %	35 %	34.3 %	€336	[6]
CIN II	98 %	55 %	53.9 %	€336	
CIN III	97 %	55 %	53.3 %	€1,498	
Cervical cancer	100 %	76 %	76 %	€12,499	
Anal cancer	87 %	79 %	68.7 %	Females €25,097; males €29,473	[4]
Vulvar cancer	100 %	37 %	37 %	€12,499	(Equal to cervical cancer cost)
Vaginal cancer	100 %	61 %	61 %	€12,499	
H&N cancer (in sensitivity analysis)	78-96 % on persistent infection	19 %	18.2 %	Females €16,990; males €18,188	[5]

^a^Sources for vaccine efficacy. Gardasil SPC; in the absence of clinical data on vaccine’s efficacy against H&N cancers (for which there is currently no indication), efficacy against HPV16/18 persistent infection has been used in sensitivity analysis (no efficacy considered in base case)

^b^Sources for proportion attributable to HPV 6/11/16/18, [27–33, 1, 34]

### Economic modelling and appraisals

The age-specific incident cases of GW, CIN and HPV-related cancers were multiplied by the, per case, medical costs. The age and disease specific deaths were multiplied by the projected loss of earnings and tax revenue for the remaining statistical life. The above two were summed to estimate the burden of disease (BOD) with and without immunization for the society and the government (i.e. societal BOD and fiscal BOD models).

Firstly, a fiscal cost-benefit analysis (CBA) was conducted following a “government perspective”. The gross lifetime tax revenue loss, as a result of HPV-related deaths, of the immunized and non-immunized cohorts of males and females was projected. The projected lifetime tax revenue loss as a result of HPV-related deaths and the HPV–related health care costs were considered a measure of the fiscal BOD. The incremental BOD between the immunized and non-immunized cohort of males and females was considered as the benefit of vaccination which was subsequently compared with the cost of vaccinations to establish the net benefit [i.e. Benefits of vaccination – vaccination investment] and benefit cost ratio (BCR).

A societal CBA was also conducted based on the societal BOD difference for the immunized and the non-immunized cohorts. The societal analysis took into account the foregone consumers surplus as a result of premature mortality. Foregone surplus refers to the productivity loss, in terms of lost earnings, for the remaining statistical life of individuals that die as a result of HPV infection. Thus, in the societal analysis in order to calculate the societal BOD, HPV-related deaths were multiplied by the present value of the remaining lifetime earnings lost due to premature deaths. The societal BOD included the HPV-related medical costs as a result of the disease and the productivity loss as a result of premature deaths. In the above CBAs, a positive benefit minus cost difference and a BCR greater than one signifies a positive economic effect for vaccination.

Then, a government perspective analysis was conducted applying the “generational accounting” methodology to calculate the incremental net discounted tax (lifetime gross tax minus the lifetime transfers) [11]. This analysis helps quantifying the net effect or the net fiscal benefit for the government after taking into account the above opposing fiscal forces. In the net discounted tax analysis the immunized cohorts were expected to have increased health care costs compared to non-immunized cohorts at the beginning of life due to vaccination costs; lower lifetime healthcare costs due to prevention of HPV-related medical costs; higher life years and productive life year lived, thus more tax revenue paid to government and higher transfer costs as more people will survive to receive pensions and other allowances.

The net discounted tax analysis uses a longitudinal timeframe that constructs the average life course for immunized and non-immunized cohorts. The model simulates how the cohorts influence fiscal accounts both in terms of lifetime taxes and government transfers received based on changes in morbidity and mortality attributed to HPV vaccination. The modified “generational accounting” framework applied here combines the three modelling approaches mentioned above to capture the influence of HPV vaccination on net discounted tax for government. A positive incremental net discounted tax signifies that the government has a benefit from vaccination after taking into account the additional transfer costs associated with increased survival. The mathematical details of the aforementioned economic appraisals are illustrated in the Additional file 1: Technical appendix.

### Data inputs

The cohort size of 12 year olds was set equal to the size (n = 400,000) used in previous economic analyses of HPV vaccination in Germany [6]. Evidence from the literature was obtained in order to simulate the age- and gender-specific mortality associated with each of the HPV-related cancers. Age-specific incidences were obtained from the literature [12–14]. In the absence of age-specific mortality data for all HPV-related cancers, except for cervical cancer, age-specific mortality was modeled as a percentage of the annual incident cases dying. The case fatality rates by disease were obtained from the GLOBOCAN IARC database for Germany [12]. The epidemiological inputs in the model are described in the Additional file 1: Technical appendix.

Vaccine efficacy was obtained from the clinical trials of the quadrivalent HPV vaccine (Gardasil SmPC). The efficacy of the vaccine was weighted for the mortality and morbidity attributable to the HPV serotypes included in the vaccine i.e. HPV6/11/16/18 (Table 1). The cost for the two doses of the quadrivalent HPV vaccination was modeled at €244 VAT excluded (2 doses × €135.75 minus 19 % VAT + €15.10 administration cost) with a coverage rate of 55 % consistent with published economic analysis in Germany [6]. Cost per case estimates for GW, CIN and HPV-related cancers were also obtained from the literature [6, 2, 4, 5]. Vaccine efficacy and cost data are illustrated in Table 1.

To conduct the aforementioned economic analyses, labor market lifetime outcomes based on expected norms were obtained. Age-specific wages and age and gender specific unemployment rates for the German male and female population were quantified annually in the model based from published sources [10]. In order to account for gender differences, an estimate of the gender wage gap in Germany (23 %) was applied across all age-groups [15]. Retirement age was set at 67 years of age. Consistent with the generational accounting methodology [11].

Similarly, age-specific transfers, tax burden and health-care costs originated from the national statistics [10]. The transfers included all the documented transfers of allowances and benefits from the government to the average German individual excluding health costs and education. In the absence of gender -specific data, the same figures were used for both males and females. An average tax burden rate of 55 % was modeled to quantify direct income tax, indirect tax and social insurance contributions for the average German individual [16]. Taxes included in the analyses encompassed the tax burden of the average German citizen. Governmental transfers included benefits, allowances and subsidies received by the average German citizen from the government [10]. Health costs were inflated at an annual historical average rate of 2.4 % [10]. To reflect changes in productivity the average annual increase of the labor unit cost equal to 0.6 % was used [10]. In the base-case analysis a 1.4 % discount rate was used following the ten-year long-term bond rates. The latter was deemed as a reasonable assumption for the opportunity costs of money, since other public investments are very likely assessed under the same assumption.

One-way sensitivity analyses were performed to evaluate the impact of parameters uncertainty on the model results. The model performed a Min-Max (±20 %) sensitivity analysis to most parameters modeled as well as a variation around discount, inflation and wage productivity rates. Additional analyses were conducted to evaluate a scenario considering a 3 doses vaccine course (previous vaccination schedule) and inclusion of head and neck cancers and sick days lost based on reported data for Germany [5].

## Results

In the base-case analysis, over the lifetime of the male and female cohorts, the analysis demonstrated that vaccination against HPV (with a coverage rate of 55 %) prevented 857 HPV-related cancer deaths in Germany. Vaccination also prevented 1527 cervical cancer cases, 286 anal cancer cases, 228 vaginal cancer cases and 116 vulvar cancer cases. In addition, vaccination resulted in 45,809 less cases of genital warts and 127,464 cases of CIN I-III for the lifetime of the cohort.

For the combined population of females and males the results showed that HPV vaccination resulted in reducing both the fiscal BOD in terms of the present value of lifetime gross tax loss due to premature mortality and the societal BOD in terms of the present value of lifetime earnings or productivity loss due to premature mortality. In addition, vaccination resulted in the reduction of HPV-related medical costs (Table 2).

**Table 2:** Estimated discounted lifetime societal, fiscal and medical cost burden for immunized and non-immunized cohorts of males and females

Outcome	Immunized	Non-immunized	Incremental
BOD-fiscal in terms of discounted lifetime gross tax loss due to premature mortality	€ 117,502,550	€ 130,864,666	-€ 13,362,116
BOD-societal in terms of discounted lifetime productivity loss due to premature mortality	€ 209,825,982	€ 233,686,903	-€ 23,860,921
Discounted lifetime HPV– related medical costs	€ 692,164,462	€ 863,973,983	-€ 171,809,521
Vaccination investment cost	€107,525,880	€ -	€ 107,525,880

When comparing the investment costs and the benefits of vaccination from a government perspective, the fiscal CBA suggested that €1 invested in universal HPV returns €1.7 in terms of averted tax revenue loss and prevented HPV-related medical costs. Female vaccination yielded, as expected, higher returns compared to male vaccination. Investing in male vaccination may offset almost one third of the investment costs whereas, for females the returns are 3-fold the investment cost (Table 3). Thus, the results suggest that there are fiscal gains associated with vaccinating females which counterbalance the cost of immunizing males. In the societal CBA, it was estimated that investment in vaccination results in BCR of 1.8 hence, in positive returns in terms of averted lifetime productivity loss and prevented HPV-related medical costs.

**Table 3:** CBA of male, female and universal vaccination from the government and societal perspectives

Societal CBA	Discounted benefits minus Investment Costs	Discounted BCR
Male	€ (37,465,618)	0.3
Female	€ 125,610,180	3.3
Both	€ 88,144,562	1.8
Fiscal CBA	Discounted benefits minus Investment Costs	Discounted BCR
Male	€ (37,858,997)	0.3
Female	€ 115,504,754	3.1
Both	€ 77,645,757	1.7

### Net discounted tax analysis

**Fig. 1:**
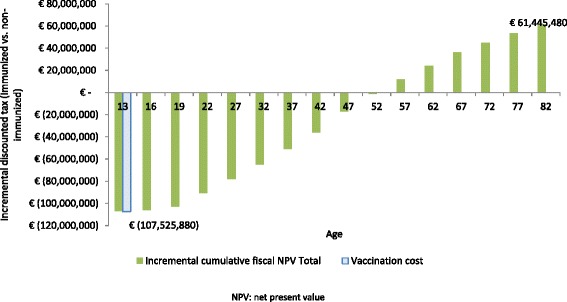
Cumulative incremental net discounted tax of the combined cohort over time. NPV: net present value

Figure 1 provides a time series for the incremental net discounted tax (tax minus transfers) between immunized and non-immunized cohorts, respectively. Universal HPV immunization could result in incremental positive net discounted taxes of €61 million for the German government. The results of the combined net discounted tax analysis suggest that even after deducting transfers, the German government has a fiscal benefit from immunizing the female and male population.

### One-way sensitivity and scenario analysis

**Fig. 2:**
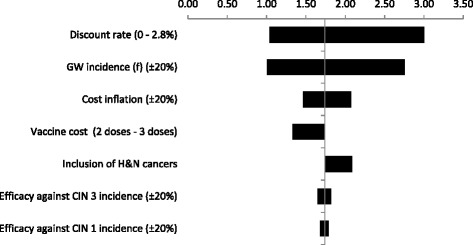
One-way sensitivity and scenario analyses for the fiscal BCR

We hereby present the tornado diagrams (Fig. 2) for the most sensitive parameters and the scenarios run, for the fiscal or gross tax based CBA. The results of the sensitivity analysis suggested that a number of economic variables were associated with high sensitivity however; the BCR consistently remained above 1 except in one scenario. One noteworthy finding was the influence of the incidence of GW which, highlights the economic importance of this outcome. The reason for the high sensitivity may relate to the high incidence of GW as well as the accumulation of most GW cases in early years of life. Since this is a discounted flows’ analysis, the selection of discount rate has a high impact on the final results as well as the rate for inflating medical and other costs in the future.

The sensitivity analysis results showed that a variation of +/− 20 % in the vaccine cost (vaccine price plus administration cost) resulted in positive fiscal BCR (>1). If three doses of vaccine were administered the BCR would decrease to 1.3. An additional scenario included the indirect costs of HPV related diseases in terms of sick days lost. As expected the results are more favorable for the vaccination compared to the basic scenario. Finally, the scenario including vaccine potential impact against head and neck cancers (for which the vaccine has currently no indication) resulted in increasing the fiscal BCR from 1.7 to 2. The latter influence of the results can be explained from the reduction of male mortality and the relatively higher lifetime earnings of males.

## Discussion

Previous recommendations by the German Standing Committee on Vaccinations have found that vaccinating adolescent females against HPV is a cost-effective option in Germany [2]. Moreover, a recent study estimating the clinical benefits of vaccinating males and females suggested that in addition to clinical benefits, substantial economic benefits are anticipated from adding males into immunization programs against HPV [17]. Conventional cost-effectiveness analyses typically focus on the health service costs and benefits and may not address the broader benefits of vaccination by defining how government and society in general will benefit from investing in vaccines.

The distinction in the analysis described here is that we consider tax financed health systems to be within the government sphere; hence changes in HPV related morbidity and mortality can influence government transfers. This analysis demonstrates that investments in HPV universal immunization yield positive benefits for government in terms of cost savings and increased tax revenue. The fiscal CBA estimated that investing in universal HPV vaccination could generate a positive rate of return, equal to a BCR of 1.7 for every Euro spent on immunization by the German government. Moreover, this study showed that from a societal perspective, surplus societal benefits (in terms of additional earnings) could be achieved by vaccinating males and females against HPV.

In recent years, preventative healthcare budgets, which account for approximately 3 % of total healthcare expenditures in Europe have been decreasing, suggesting a need to highlight the health and economic importance of prevention [18]. Indeed, when positioned within the broader consequences of ageing populations, the benefits of preventative healthcare for influencing working-aged populations is likely to gain increasing attention [19]. In 2009, the World Health Organization [20] developed the WHO Guide to Identify the Economic Consequences of Disease and Injury, in which the impact of poor health on government fiscal accounts was recognized. In contrast to documentation from the WHO regarding broader economic impact of health, local funding decisions in health-care are mostly based on clinical and cost-effectiveness measures, and metrics such as productivity and economic growth are given only cursory attention.

The analysis described here applies a lifetime horizon and financial metrics to estimate fiscal consequences associated with HPV immunization and suggests that, over a lifetime span, the vaccination of males and females offers positive fiscal benefits. There are many inherent weaknesses in making long-term projections based on current macroeconomic parameters due to uncertainty regarding the future. In this respect, the results described here should not be seen as a precise forecast of the future. Rather, the fiscal analysis reflects a potential scenario based on current economic and epidemiological conditions, that future changes in unemployment, growth rates, tax burden, government transfers or inflation could either positively or negatively influence the findings described here. This is particularly important to consider in relation to the static incidence rates applied in our analysis and failure to account for herd immunity which could influence incidence rates and the likely benefits attributed to vaccination. Consistent with the generational accounting framework on which our analysis is based, we have held parameters constant over time. We acknowledge this weakness but in the absence of knowledge of the future, the approach is useful for making policy decisions today, and, in this respect, is not different than any other funding or policy decisions made by governments.

In addition, the modelled mortality and morbidity data in this study, do not take into account socio-economic status which may influence HPV incidence and mortality and treatment seeking patterns that, in turn, may affect labour productivity and tax revenue loss as measured by the human capital and generational accounting methods, respectively. Moreover, with the exception of cervical cancer, case-fatality rates were used to estimate age-specific mortality. It should also be noted that the results presented in this study are specific to the German macroeconomic setting. Given cross-country differences in macroeconomic parameters and the health system production functions, it is expected that the benefits from HPV immunization vary across countries. Further research should focus on the generalizability of the results to other country-settings.

Probabilistic sensitivity analysis (PSA) is commonly applied in cost-effectiveness analysis to inform healthcare stakeholders of parameter uncertainty. Because our modelling approach does not follow the conventional “health service” cost-effectiveness perspective, it seeks to inform an audience within government about the fiscal impact of investing in immunization programs, and epidemiological outcomes have been translated into fiscal gains and losses for government. The model is based on a modified generational accounting framework which informs government about costs and consequences of investments in technology. As PSA is not a feature of this modelling approach, and not relevant to the target audience for this work, it has not been applied in our analysis. It is conceivable that PSA could be applied however, in the absence of distributions for each variable, arbitrary ranges can only be used which may provide limited uncertainty analysis outcomes. The univariate sensitivity analysis applied in this model was deemed as adequate for both understanding key areas of uncertainty, and informing stakeholders of the potential gains and losses from investing in HPV vaccination.

Decision-making regarding funding of vaccines is a complex process involving numerous government stakeholders and health service officers. Even when vaccines have been approved by national advisory groups, it is acknowledged that funding should be independently sought from finance ministries [21]. Underpinning the importance of finance in the decision-making process, some have advocated that finance ministries should be involved earlier in the consultation process to ensure more rapid uptake of vaccines [22]. To this end, it is important to provide financiers with economic metrics which they are familiar with, to demonstrate the relationship between vaccination, health and growth. By generating measures typically used in the appraisals of public investments (i.e. NPV and rate of return), health interventions could be compared to other publically funded investments. Hence, the illustrated framework could be used in informing cross-sectorial resource allocation decisions.

Considering the broader economic consequences of vaccine preventable conditions suggests that changing population health through vaccination can offer economic advantages for government in the form of future tax revenues and potential savings attributed to social care. Consequently, investing in vaccination can be viewed as a public investment stimulating economic growth and influencing government finances [23, 24]. Taking into consideration the fact that many health technology appraisal agencies usually focus on a healthcare payer perspective, and do not consider indirect costs, and the resulting effects on productivity, poses additional challenges for vaccine technologies which prevent morbid events and early mortality over many generations [25].

## Conclusion

The framework described here estimates how saving lives and influencing HPV infections in males and females can generate fiscal benefits for government. This raises questions about how to apply a fiscal accounting framework in healthcare decision-making when healthcare resources are often influenced by unmet need, burden of illness, affordability, equity and sometimes politics. While it is tempting to only invest in those strategies that yield benefits for government, it is questionable whether it is justifiable to exclude treatment from some groups on the basis of having a low fiscal return for government. Clearly this is not the case as resources are allocated for many interventions in terminal conditions that generate no fiscal benefits for government. On this basis, we do not expect decision-makers to abandon the core principles of decision-making which include unmet need, equity and fairness. However, this approach can be supplementary to existing analytic frameworks used by decision-makers to consider a broader range of benefits from the perspective of government. This can be particularly important for tax financed health systems that rely on younger, healthy generations to continue paying for the healthcare needs of older generations [26].

## Additional file

Additional file 1:
**Technical appendix.**
