# Supplementary material for: Quantifying the broader economic consequences of quadrivalent human papillomavirus (HPV) vaccination in Germany applying a government perspective framework
Source: Health Econ Rev. 2015 Jul 22;5:23. doi: 10.1186/s13561-015-0054-6 (PMC4510306; doi:10.1186/s13561-015-0054-6)
Supplement: Additional file 1: — Technical appendix. [file 13561_2015_54_MOESM1_ESM.docx]

**Technical appendix**

Epidemiological calculations

Figure A1 shows the structure of the prospective single cohort model developed. Equations 1 -3 illustrate the incidence and mortality calculations included in the model for HPV related diseases. Figures A2 and A3 illustrate the age-specific epidemiology per 100,000 used in the model. It should be noted that the incidence and mortality rates used, referred to the total burden of the under study diseases (HPV-related diseases and cancers). Weighting for the proportion of incident cases and deaths that can be causally attributed to HPV infection was included in the calculation of vaccine efficacy. Thus, figures A2 and A3 illustrate the total epidemiological burden of the under study diseases rather than the proportion that can be causally linked to HPV infection.

For cervical cancer age-specific mortality data were used to simulate the annual age-specific mortality that can be attributed to cancers linked with HPV infection (equation 2). For all other cancers, annual mortality of incident cases was calculated based on case-fatality rates multiplied by the annual age-specific incidence as in equation 3. The resulting deaths at each year were summed to produce the total mortality for the under study cancers for the cohort under analysis. The total number of deaths attributed to HPV related diseases was calculated as the lifetime sum of the single cohort annual deaths that can be attributed to cervical cancer and other HPV related cancers (equations 2 and 3).

For the immunized cohort, incidence was reduced by the efficacy of the quadrivalent HPV vaccine which, in turn, was a function of vaccination coverage and the proportion of cancers that can be causally attributed to HPV infection (equation 4). Similarly, the aforementioned vaccine efficacy was used to calculate the number of cervical cancer and all-other HPV-related cancers deaths for the immunized cohort.

**Annual incidence of HPV-related diseases** = cohort’s survivors (in each year) x annual incidence of HPV related diseases

(1)

**Annual number of deaths attributable to cervical cancer** = cohort’s survivors (in each year) x age-specific mortality of cervical cancer

(2)

**Annual number of deaths attributable to HPV-related cancers** (excluding cervical cancer) = cohort’s survivors (in each year) x annual incidence of HPV related cancers x case fatality rates of HPV related cancers

(3)

**Annual incidence of HPV-related diseases** (for the immunized cohort) = cohort’s survivors (in each year) x annual incidence of HPV related disease x (1- Efficacy of the quadrivalent HPV vaccine against the incidence of HPV related epidemiology, adjusted for the proportion attributed to the vaccine HPV serotypes) x vaccination coverage

(4)

Economic appraisals

The fiscal or government perspective economic appraisal was conducted as illustrated in equations 5 and 6. Furthermore, a cost: benefit ratio (BCR) was calculated as a proxy measure of return on investment (equation 7). Annual direct medical costs were calculated as the annual number of incident HPV related disease cases multiplied by the annual cost per case. Potential side-effects associated with the vaccine were not included in this analysis as they were not expected to significantly influence the economic appraisals. In the sensitivity analysis national insurance costs were estimated based on the cost of abseentism covered by the national insurance system for the economically active individuals. Cost and benefits were discounted (r: discount rate).

The societal analysis was conducted following a similar approach however, instead of the expected annual gross tax, the expected annual earnings was factored into the calculations. In addition, in the sensitivity analysis, the societal analysis included abseentism, valued with the human capital method, as an indirect cost to the society. Thus, BOD for immunized and non-immunized cohorts reflected the societal BOD.

${BOD}_{fiscal}=\sum_{t}^{Le} \frac{expected annual gross tax}{{(1+r)}^{t}}+\sum_{t}^{Le} \frac{annual direct medical costs+(annual National Insurance costs)}{{(1+r)}^{t}}$ (5)

Net Benefit fiscal = (BOD _fiscal non-immunized_ - BOD _fiscal immunized_) – Vaccination cost (6)

BCR fiscal = (BOD _fiscal non-immunized_ - BOD _fiscal immunized_) : Vaccination cost (7)

The generational accounting assessment of net discounted tax was assessed using equations 8-10.

${Net discounted tax}_{lifetime}= \sum_{13}^{Le} \frac{{Tax}_{t}-{Cost}_{t}}{{(1+r)}^{t}}$ (8)

Where in year of age t:

${Tax}_{t}={Direct tax}_{t}+{Indirect tax}_{t} +{National insurance contributions}_{t}$ (9)

${Cost}_{t}= {Health care costs}_{t}+{Transfers}_{t} +{Pension}_{t}$ (10)

**Figure A1**

**Modelled epidemiological inputs**

**Figure A2**

**Figure A3**

*Kraut et al., 2010; ** Siebert et al., 2006; ^+^IARC Globocan, 2008
